# The long term microbiota and metabolic status in patients with colorectal cancer after curative colon surgery

**DOI:** 10.1371/journal.pone.0218436

**Published:** 2019-06-14

**Authors:** Xi-Hsuan Lin, Jeng-Kai Jiang, Jiing-Chyuan Luo, Chung-Chi Lin, Po-Hsiang Ting, Ueng-Cheng Yang, Yuan-Tzu Lan, Yi-Hsiang Huang, Ming-Chih Hou, Fa-Yauh Lee

**Affiliations:** 1 Department of Medicine, School of Medicine, National Yang Ming University, Taipei, Taiwan; 2 Division of Gastroenterology and Hepatology, Department of Medicine, Taipei Veterans General Hospital, Taipei, Taiwan; 3 Department of Surgery, School of Medicine, National Yang Ming University, Taipei, Taiwan; 4 Division of Colorectal Surgery, Department of Surgery, Taipei Veterans General Hospital, Taipei, Taiwan; 5 Healthcare and Management Center, Taipei Veterans General Hospital, Taipei, Taiwan; 6 Institute of Biomedical Informatics, National Yang Ming University, Taipei, Taiwan; State Key Laboratory for Diagnosis and Treatment of Infectious Diseases, CHINA

## Abstract

Whether there are subsequent changes of metabolic profiles and microbiota status after partial colectomy remains unknown. We evaluated and compared long-term effects of microbiota status and metabolic profiles in early colorectal cancer (CRC) patients after curative colectomy to the controls. In this cross-sectional study, we analyzed metabolic syndrome occurrence in 165 patients after curative partial colectomy with right hemicolectomy (RH) or low anterior resection (LAR) and 333 age-sex matched controls. Fecal samples from some of those with RH, LAR, and controls were analyzed by next-generation sequencing method. The occurrences of metabolic syndrome were significantly higher in patients after RH, but not LAR, when compared with the controls over the long term (> 5 years) follow-up (P *=* 0.020). Compared with control group, RH group showed lower bacterial diversity (P = 0.007), whereas LAR group showed significantly higher bacterial diversity at the genera level (P = 0.016). Compared with the control group, the principal component analysis revealed significant differences in bacterial genera abundance after RH and LAR (P < 0.001). Furthermore, the Firmicutes to Bacteroidetes ratio was significantly lower in the RH group than the control group (22.0% versus 49.4%, P < 0.05). In conclusion, early CRC patients after RH but not LAR were associated with a higher occurrence of metabolic syndrome than the controls during long-term follow-up. In parallel with metabolic change, patients with RH showed dysbiosis with a tendency to decreased richness and a significant decrease in the diversity of gut microbiota.

## Introduction

The gastrointestinal (GI) tract plays an important role in nutritional homeostasis and metabolic control [1]. Bariatric surgery which modified upper GI tract structure and function has widely demonstrated a beneficial effect on obesity and type II diabetes mellitus [2,3]. However, there was little literature focused on the relationship between lower GI tract and metabolic control. Study showed that patients with loss of the colon after surgical resection exhibit some characteristics of the insulin resistance [4,5].

The mortality and incidence related to colorectal cancer (CRC) were rising in Asia [6]. Surgery offers the hope of cure in most patients with CRC. At present, accumulating evidence demonstrates that gut microbiota played an important role in the initiation and development of CRC [7,8]. Moreover, it has gained more attention about the role of the gut microbiota and microbial metabolites in the development of metabolic disease including obesity and metabolic syndrome [9]. However, it is not known whether there are further changes of metabolic profiles and microbiota status after partial colectomy for curable CRC. As a result, understanding the consequences for partial colectomy is not only an important issue for these patients, but also gives an insight into the crosstalk between the microbiota and metabolic diseases.

In line with this thinking, we aimed to investigate and compare long-term effects of metabolic profiles and microbiota status in CRC cancer patients receiving curative partial colectomy to the controls.

## Materials and methods

### Study population

We performed a cross-sectional study at Taipei Veterans General Hospital. 165 subjects at outpatient clinics successively who had underwent curative surgery including right hemicolectomy (RH) or low anterior resection (LAR) for primary nondisseminated CRC in Taipei Veterans General Hospital between 2006–2010 were identified and enrolled in our study group. The time of follow-up after surgery (RH or LAR) to study enrollment was 8.75 years (ranging 7.00 to 10.5 years). To ensure adequate long term follow-up, patients who had underwent curative surgery after 2010 were excluded. Patients with the following conditions were also excluded: 1.) aged less than 20 years; 2.) underlying other malignancies; 3.) pre- and postoperative chemotherapy or chemoradiotherapy for CRC; 4.) other endocrine disorders such as diabetes mellitus, thyroid, pituitary or adrenal disease; 5.) moderate to severe cardiovascular, pulmonary, hepatic, or renal disease; 6.) recurrent CRC after curative surgery. Furthermore, patients who had received proton pump inhibitors, histamine-2 receptor antagonists, nonsteroidal anti-inflammatory drugs, antibiotics, or probiotics within 1 month of sample collection were excluded. In addition, 1:2 age-sex, time of follow-up matched subjects without surgery for GI tract were also enrolled as the control group with the same exclusion criteria as study group. Totally 333 subjects were enrolled as the controls.This study complies with the standards of the Declaration of Helsinki and current ethical guidelines and has been approved by the hospital’s Institutional Review Board (IRB)(2016-07-008B).

### Anthropometric and laboratory measurement

After signing informed consents, anthropometric measurements (i.e., body mass index [BMI], waist circumference, and blood pressure [BP]) were taken by experienced nursing staff. Blood tests including serum fasting glucose, total cholesterol, high-density lipoprotein (HDL), triglyceride (TG) were collected after an overnight fast. Metabolic syndrome was measured and diagnosed if three or more criteria were met: 1.) abdominal obesity, waist circumference 90 cm in males and ≥80 cm in females; 2.) high blood pressure, ≥130 mmHg systolic, ≥85 mmHg diastolic, or current medication for hypertension; 3.) high serum fasting glucose, ≥100 mg/dL or current use of anti-diabetic therapy; 4.) low high-density lipoprotein (HDL)-cholesterol <40 mg/dL in males and <50mg/dL in females; and 5.) hypertriglyceridemia ≥150mg/dL [10,11].

### Stool bacterial genomic DNA extraction and PCR amplification

We further analyzed anthropometric, laboratory, and fecal microbiome from 10 patients who had undergone partial colectomy with RH, 10 study patients with LAR, and 20 controls of the same study subjects. Fresh stool samples were collected at home at the same day or one day before blood measurement and immediately frozen in their home freezers at −20°C which were then delivered to the hospital within four hours in insulating polystyrene foam containers and stored at −80°C until DNA extraction. Overall, the mean time interval between surgery and measurement was 8.5 years.

Bacterial genomic microbial DNAs were extracted using the QIAamp DNA Stool Mini Kit (Qiagene, MD, USA) according to the manufacturer’s protocols. Briefly, tissue samples (180~220 mg) yielded 5–100 μg genomic DNA for direct use in 16S rRNA gene sequencing. Amount and quality of isolated genomic DNA was determined with NanoDrop ND-1000 (Thermo Scientific, Wilmington, DE, USA). Genomic DNA was stored at –80°C prior to 16S rRNA sequencing. One μl of sample DNA (10 pg~500 ng) was used as template in a PCR reaction for bacteria 16S rRNA variable region V3–V4. The primer set for the reaction was chosen with 341F_V3_illumina (5′ - CCTACGGGNGGCWGCAG-3′) and 805R_V4_illumina (5′ - GACTACHVGGGTATCTAATCC -3′) [12]. PCR consisted of an initial denaturation at 94°C for 2 min, then 30 cycles of 92°C for 20 sec, 55°C for 30 sec and 68°C for 1 min for amplification, 68°C for 1 min to finish replication on all templates, stored at 4°C. Dual-indexes (barcodes) were used for each sample before sequencing and NGS was performed by the Illumina MiSeq Desktop Sequencer following the standard protocol.

### Data processing and statistical analysis

From the initial clean reads, the following steps were performed to obtain the final effective reads: Demultiplex each sample by dual-index using “in-house script”; Join paired-end reads using program “PEAR”; Trim primer sequences from joined reads using program “AlienTrimmer”; Trim off low quality end sequences by sliding windows (5 nt) with average quality value under 10 and screen out short sequences less than 200 nt using program “Trimmomatic”; Filter out chimeric reads using software package “Mothur” v.1.33.3 (Department of Microbiology & Immunology at The University of Michigan, USA).16S rDNA analysis with the Greengenes 16S rRNA Taxonomy Database (gg_13_8) was performed by the software package ‘Mothur’ version 1.33.3 and ‘QIIME’ version 1.80 [13]. We calculated the Chao richness estimator, Fisher's alpha diversity index and the Shannon diversity index (SI) using the Mothur program.

Taxonomic microbiota profiles were submitted to principal component analysis (PCA) which was performed on log-transformed data using the R package ADE4 to analyze genera abundance between groups [14]. Between-group inertia percentages was tested (Monte-Carlo test with 10000 permutations) to determine the *P*-values of PCA results. Statistically significant differences in the relative abundance of taxa associated with groups of patients were performed using linear discriminant analysis (LDA) effect size (LEfSe) with α = 0.05 (Kruskal-Wallis and Wilcoxon tests) and effect size threshold of 2 on linear discriminant analysis (LDA) through the web site, http://huttenhower.sph.harvard.edu/galaxy [15].

All data were expressed as means ± standard deviation. If some parameters were not normally distributed, nonparametric analysis was used. Results were compared between groups depending on the type of data analyzed using either Chi-square test, Fisher's Exact test, Student's t test or nonparametric Mann- Whitney U test when appropriate. All statistical analyses were performed using Sample Power release 2.0 and SPSS for Windows version 14.0 (both by SPSS Inc, Chicago, IL, USA). All P values are two-tailed, and a *p* value less than 0.05 was considered statistically significant.

## Results

### Metabolic status in patients post partial colectomy

One hundred and sixty five patients in our study group and 333 age-sex matched subjects in our control group were enrolled to investigate the long-term effects of partial colectomy on the metabolic profile including 53 right hemicolectomy (RH) and 112 low anterior resection (LAR).

Compared with the control group, patients who received RH or LAR had higher systolic BP, higher serum fasting glucose, and higher occurrence of metabolic syndrome (*P* < 0.05) (Table 1). There were no statistically significant differences in the BMI, waist circumference, diastolic BP, serum TG, total cholesterol, and HDL level between the two groups (*P* > 0.05) (Table 1).

**Table 1:** Anthropometric and laboratory data between patients with partial colectomy and the control group with a median follow-up of 8.75 years.

	Control subjects N = 333	Patients with partial colectomy N = 165	Patients with LAR N = 112	Patients with RH N = 53
Age y/o	71.1 ± 5.0	70.1 ± 10.3	70.0 ± 10.4	71.4 ± 9.8
Sex (M: F)	191: 142	92: 73	69: 43	23: 30
body mass index	24.5 ± 3.1	24.4 ± 3.2	24.6 ± 3.0	23.8 ± 3.6
waist (cm)	86.4 ± 10.8	87.0 ± 7.6	87.8 ± 7.0	86.0 ± 8.8
systolic BP (mm Hg)	126 ± 18	130 ± 14*	130 ± 13	131 ± 16
diastolic BP (mm Hg)	75 ± 11	76 ± 12	77 ± 14	76 ± 10
HDL-cholesterol (mg/dL)	51 ± 13	51 ± 12	48 ± 10	55 ± 13
Total cholesterol (mg/dL)	190 ± 38	185 ± 32	184 ± 31	187 ± 34
Triglyceride (mg/dL)	120 ± 60	118 ± 58	115 ± 53	123 ± 68
Serum glucose (mg/dL)	100 ± 22	107 ± 22*	105 ± 20	110 ± 24*
Metabolic syndrome + (%)	104 (31.2%)	80 (48.5%)*	50 (44%)	30 (56.6%)*

GI, gastrointestinal; BP, blood pressure; HDL, high density lipoprotein; LAR, low anterior resection; RH, right hemicolectomy.

* P<0.05 when compared with the control group.

In subgroups analysis between RH patients and control group, patients who received RH had higher serum fasting glucose and higher occurrence of metabolic syndrome than those of the controls (*P* < 0.05) (Table 1). However, there were no significant differences in the BMI, waist circumference, systolic BP, diastolic BP, serum TG, total cholesterol, and HDL level between the two groups (*P* > 0.05) (Table 1). When compared with the controls, there were no significant differences in the BMI, waist circumference, systolic BP, diastolic BP, serum fasting glucose, serum TG, total cholesterol, and HDL level as well as the occurrence of metabolic syndrome between LAR group and the control groups (*P* > 0.05) (Table 1).

To investigate the long-term effects of partial colectomy on the gut microbiota in CRC patients, we further analyzed anthropometric, laboratory, and fecal microbiome from 10 patients who had undergone partial colectomy with RH, 10 study patients with LAR, and 20 controls of the same study subjects. Compared with the control group, patients who had undergone partial colectomy with RH had a trend to have higher serum fasting glucose and higher occurrence of metabolic syndrome (*P* all < 0.15) (Table 2). However, when compared with the controls, there were no significant differences or trends between LAR group and the control groups (Table 2).

**Table 2:** Anthropometric and laboratory data between patients with partial colectomy (right hemicolectomy or low anterior resection) and controls with fecal microbiome.

	Control subjects N = 20	Patients with LAR N = 10	Patients with RH N = 10
Age y/o	73.1 ± 7.0	72.7 ± 7.0	74.4 ± 9.8
Sex (M: F)	12: 8	6: 4	6: 4
body mass index	23.1 ± 3.5	24.6 ± 3.0	23.5 ± 3.4
waist (cm)	85.4 ± 11.2	86.6 ± 7.0	88.9 ± 9.2
systolic BP (mm Hg)	125 ± 15	130 ± 16	129 ± 22
diastolic BP (mm Hg)	77 ± 10	80 ± 11	75 ± 9
HDL-cholesterol (mg/dL)	58 ± 11	56 ± 10	50 ± 15
Total cholesterol (mg/dL)	199 ± 31	185 ± 36	186 ± 24
Triglyceride (mg/dL)	108 ± 42	108 ± 40	109 ± 43
Serum glucose (mg/dL)	95 ± 18	95 ± 15	106 ± 12
Metabolic syndrome + (%)	7 (35%)	4 (40%)	6 (60%)

GI, gastrointestinal; BP, blood pressure; HDL, high density lipoprotein; LAR, low anterior resection; RH, right hemicolectomy.

There were no significant difference between t groups

### Statistical summaries of sequencing results

After 16S rRNA gene sequencing and quality filtering, 2.6 million reads from a total of 3.4 million pair-end reads were obtained corresponding to a mean of 65±19 thousand reads per sample. (S1 Table)

### Richness and diversity of gut microbiota

**Fig 1:**
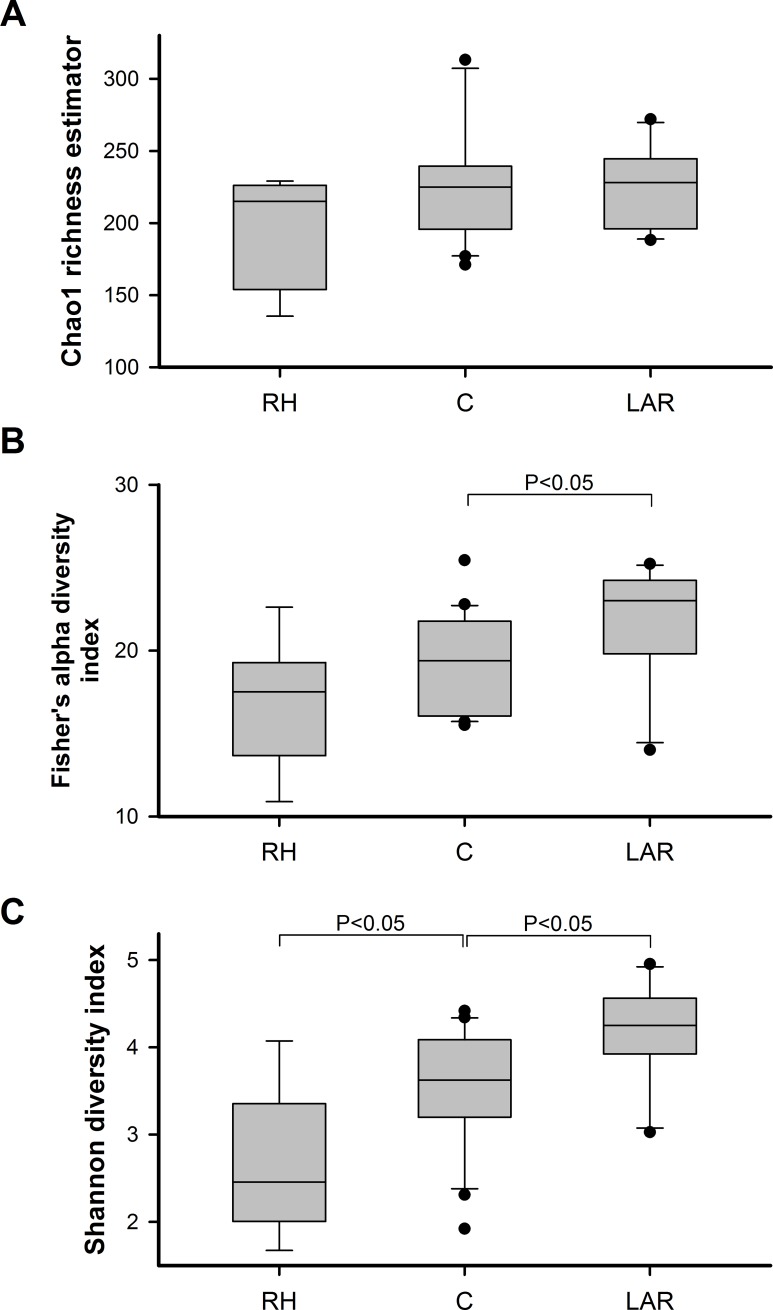
Richness and diversity of gut microbiota between patients with partial colectomy (right hemicolectomy or low anterior resection) and controls. Chao1 estimated no significant difference among RH, LAR, and control group though a tendency to decrease in bacterial richness in RH group when compared with others (Fig 1A). LAR group showed higher bacterial diversity, as estimated by the fisher's alpha diversity index and Shannon diversity index, when compared with control group (p < 0.05, Fig 1B & 1C). RH group showed lower bacterial diversity, as estimated by the Shannon diversity index when compared with control group (p < 0.05, Fig 1C). The boxes (containing 50% of all values) show the median (horizontal line across the middle of the box) and the interquartile range, whereas the black spots represent the 10th and the 90th percentiles.

The gut microbiota richness was estimated by Chao (Fig 1A and S2 Table). Compared with the control group, RH group showed a tendency to decrease the richness at the genera level (Chao, *P* = 0.105), LAR group showed no difference in bacterial richness at the genera level (Chao, *P* = 0.620).

The fisher's alpha diversity index and Shannon diversity index (SI) were used to evaluate the ecological diversity of microbiota from each sample (Fig 1B and 1C and S2 Table). Compared with control group, RH group showed a tendency to decrease the fisher's alpha diversity (*P* = 0.116), whereas LAR group showed significantly higher bacterial diversity index at the genera level (*P* = 0.039). Compared with control group, RH group showed lower Shannon diversity (*P* = 0.007), whereas LAR group showed significantly higher Shannon diversity at the genera level (*P* = 0.016).

### Long-term effects on gut microbiota composition

**Fig 2:**
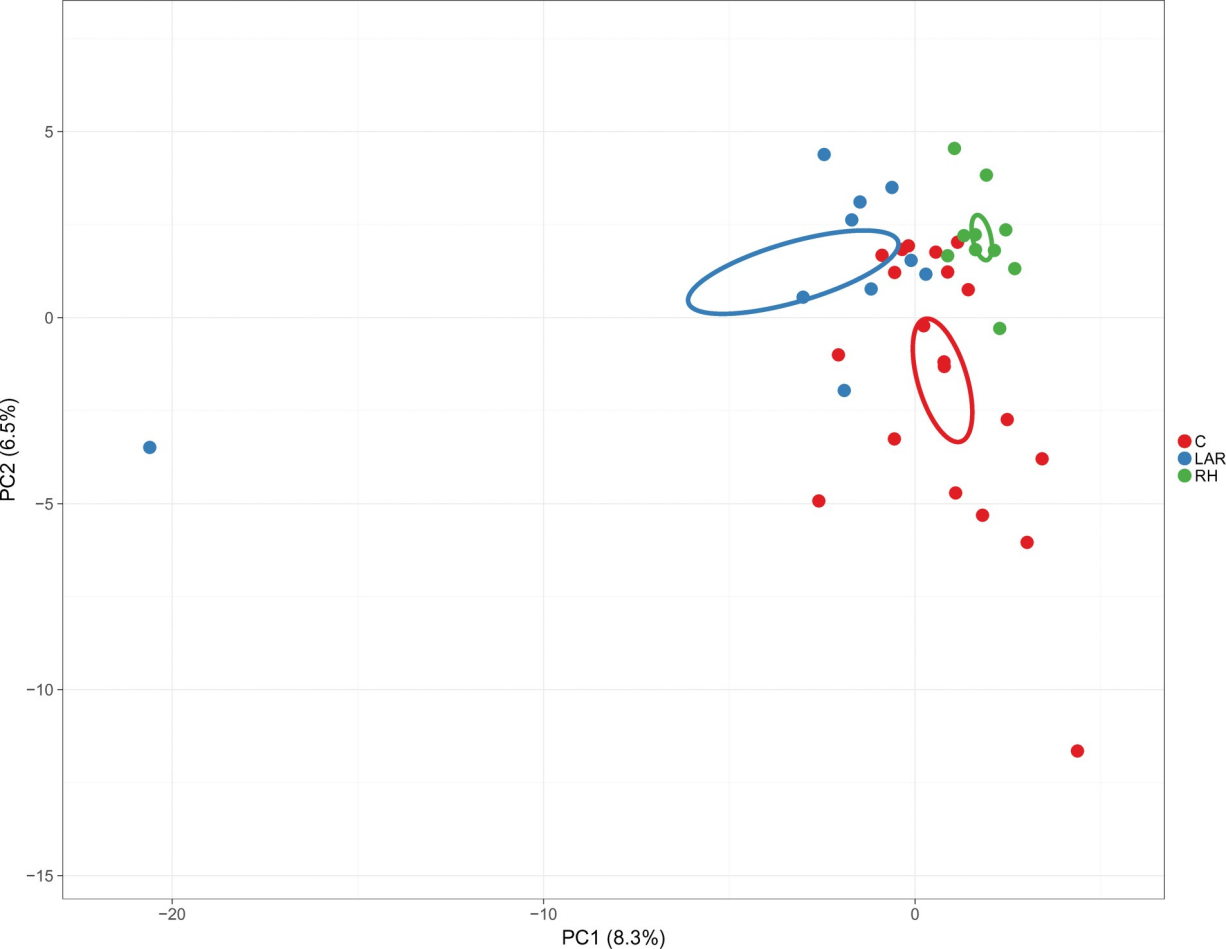
Principal component analysis of bacterial genera abundance. **G**ut microbial genera composition using a principal component analysis (PCA) of the log-transformed relative abundances showed a clear separation between control group, and those after right hemicolectomy (RH) or low anterior resection (LAR). Compared with control group, RH group and LAR group revealed significant differences in bacterial genera abundance respectively (p all < 0.001, Monte-Carlo simulation).

We visualized the changes in overall gut microbial genera composition using a PCA of the log-transformed relative abundances (Fig 2), which showed a clear separation between control group, and those after RH or LAR. Compared with control group, PCA revealed significant differences in bacterial genera abundance after RH (*P* < 0.001, Monte-Carlo simulation; Fig 2) and LAR (*P* < 0.001, Monte-Carlo simulation; Fig 2).

**Fig 3:**
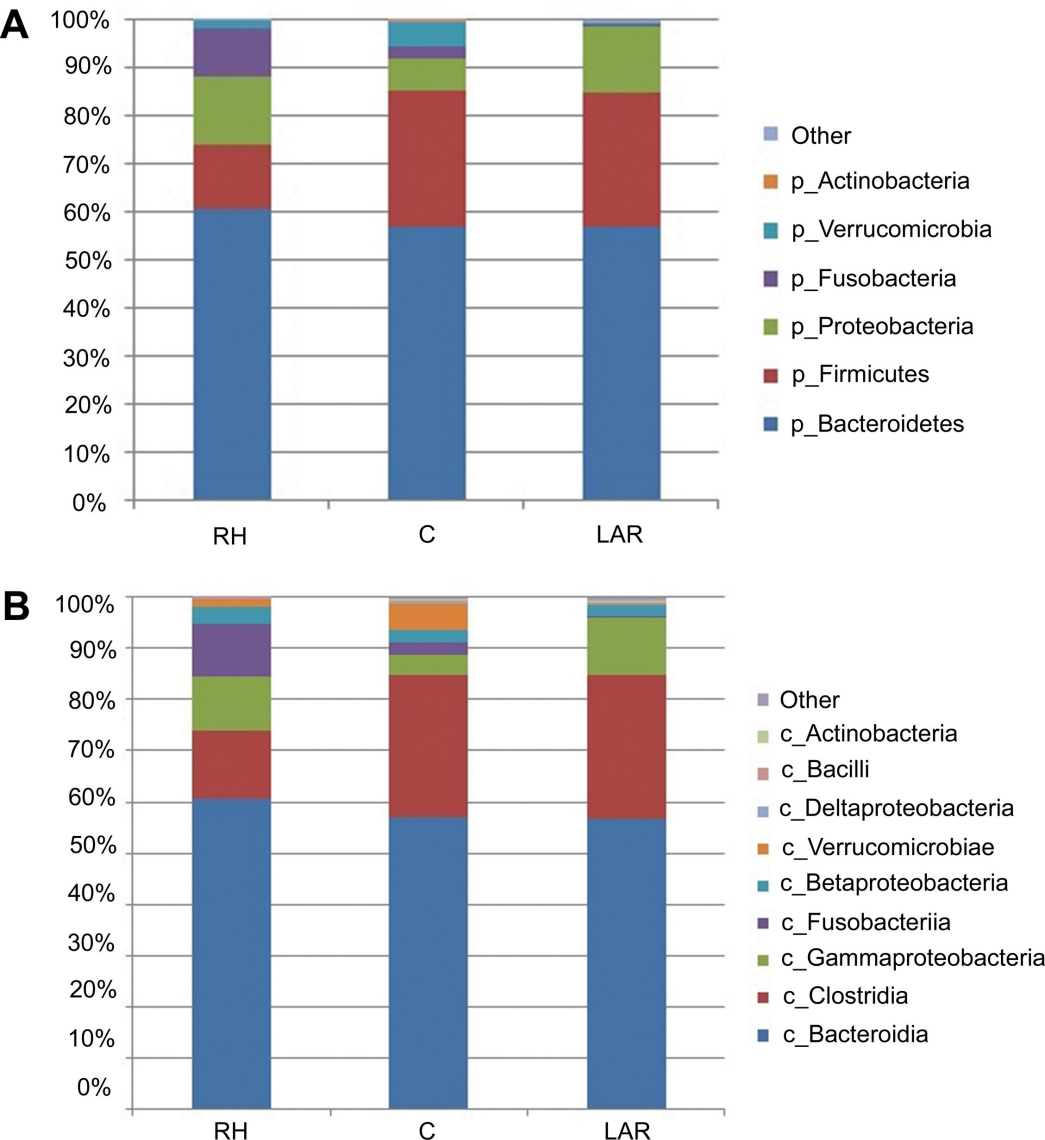
Relative abundances of classes across three groups. Relative abundances of phylum across right hemicolectomy (RH), low anterior resection (LAR), and control groups (Fig 3A). Relative abundances of classes across right hemicolectomy (RH), low anterior resection (LAR), and control groups (Fig 3B).

The four major phyla in microbiota of the RH, LAR, and control groups were Bacteroidetes, Firmicutes, Proteobacteria and Fusobacteria (Fig 3A). RH patients had a higher proportion of Fusobacteria (10.3% versus 2.6%, *P* < 0.05) than controls and lower proportions of Firmicutes (13.4% versus 28.2%, *P* < 0.05) than those in controls. LAR patients had a higher proportion of Proteobacteria (13.9% versus 6.5%, *P* < 0.05) than controls. Furthermore, the Firmicutes to Bacteroidetes ratio was significantly lower in the RH patients than the control group (22.0% versus 49.4%, *P* < 0.05).

Dominant class represented ≥ 0.2% of obtained gut microbiota sequences. There were nine known dominant classes shown in Fig 3B including Bacteroidia, Clostridia, Gammaproterobacteria, Fusobacteriia, Betaproteobacteria, Verrucomicrobiae, Deltaproteobacteria, Bacilli, and Actinobacteria.

The bacterial class Fusobacteriia, belonging to Fusobacteria, was significantly higher in RH group compared with control group (10.3% vs. 2.6%, *P* < 0.05).

Moreover, compared with control group, the bacterial class Clostridia, belonging to Firmicutes, was significantly lower in RH group (13.2% vs. 27.8%, *P* < 0.05). The bacterial class Deltaproteobacteria, belonging to Proteobacteria, was significantly lower in RH group compared with control group (0.1% vs. 0.3%, *P* < 0.05). The bacterial class Gammaproteobacteria belonging to Proteobacteria, was significantly higher in LAR group compared with control group (11.% vs. 3.8%, *P* < 0.05). Moreover, compared with control group, the bacterial class Verrucomicrobiae belonging to Verrucomicrobia, was significantly lower in LAR group (0.1% vs. 5.65%, *P* < 0.05).

**Fig 4:**
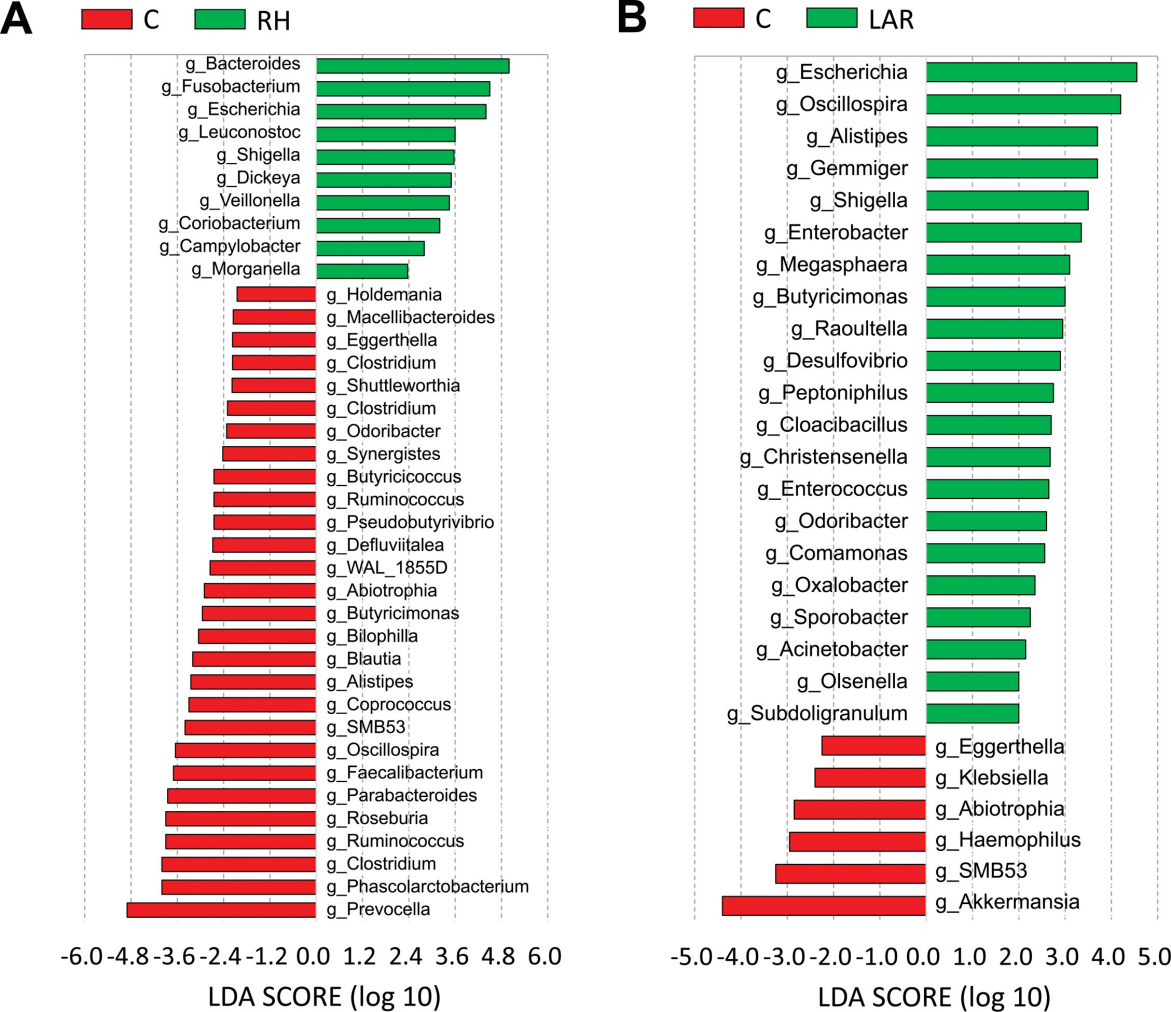
Known genera abundance reported by LEfSe in the bacterial community. Known genera reported by LEfSe in the bacterial community, comparison between right hemicolectomy (RH) and control group (Fig 4A). Known genera reported by LEfSe in the bacterial community, comparison between low anterior resection (LAR) and control (Fig 4B).

The discriminant analysis by using LEfSe approach was applied to identify the key taxa responsible for the difference between study groups. The identified taxa were highlighted on the cladogram along with their LDA scores (Fig 4). We identified a total of 38 known genera which contributed to the difference between the fecal microbiota of RH group and control group. (Fig 4A) Among 10 genera known to be more abundant in RH group, Bacteroides and Fusobacterium represented the top two genera. Interestingly, buryrate-producing bacteria including Roseburia species and Faecalibacterium prausnitzii were less abundant in RH group. There were a total of 27 known genera with significant differences between LAR group and control group (Fig 4B). Among 21 genera known to be more abundant in LAR group, Escherichia and Oscillospira represented the top two genera.

## Discussion

In our study, we found the long-term metabolic change including higher fasting serum glucose and higher occurrence of metabolic syndrome in CRC patients after partial colectomy. Our findings are comparable to previous studies which reported that concomitant hyperglycemia and hyperinsulinemia, the key feature of metabolic syndrome, were the most consistent finding following total colectomy even though the underlying pathology for colectomy including inflammatory bowel disease, Hirschsprung’s disease and CRC was different [4,5,16–18].

To the best of our knowledge, the present study is the first study to evaluate the long term metabolic change and risk of metabolic syndrome in CRC patients following partial colectomy. The explanations of higher serum fasting glucose and higher occurrence of metabolic syndrome in CRC patients after partial colectomy may be related to several potential mechanisms: 1.) the increased adipose tissue lipolysis due to reduced short-chain fatty acid (SCFA) synthesis produced from colonic fermentation [17,19], 2.) the decreased glucagon-like peptide 1 (GLP-1) concentrations secreted by endocrine L-cells in the distal gut [4,17], 3.) the secondary aldosteronism [20,21]. Furthermore, the finding that RH patients, rather than LAR patients, had a higher serum fasting glucose and higher occurrence of metabolic syndrome when compared with those of control imply that proximal colon may play an important role in glucose control. From the proximal to the distal colon, there is a decreasing trend in SCFA concentration which makes the proximal part of the colon the principal site of fermentation [22].

Given the role of the gut microbiota in regulating host metabolism, this is the first study in which deep sequencing is applied to investigate the long term effect of microbiota status in CRC patients after curative partial colectomy with RH and LAR respectively. In parallel with metabolic change including the higher serum fasting glucose and higher occurrence of metabolic syndrome, gut microbial richness and diversity decreased after RH rather than LAR. We also found an obvious shift toward a lower abundance of Firmicutes phylum and Clostridia class after RH. The finding was consistent with Larsen’s study showing that the proportions of Firmicutes phylum and Clostridia class were significantly reduced in the type II diabetic group when compared to the non-diabetic group [23]. Furthermore, the ratio of Firmicutes to Bacteroidetes was significantly lower in the RH patients than the control group in our study, which was in agreement with two previous studies consistently showing a significantly lower Firmicutes to Bacteroidetes ratio in diabetes mellitus patients compared with control group [23,24].

In our study, we found an obvious shift toward a higher abundance of Fusobacteria phylum, Fusobacteriia class and Fusobacterium genus after RH. Sakalauskiene, et al showed that the presence of Fusobacterium nucleatum, an aerobic periodontal pathogen in subgingival plaque samples was identified more frequently in the diabetic group than in the healthy group [25]. As a result, there was a strong association between the colonization of gut lumen by Fusobacterium species and the hyperglycemic status in humans.

In our study, we further found a reduction in Roseburia species and Faecalibacterium prausnitzii, both well-known butyrate-producing bacteria which belong to the Clostridia cluster of the Firmicutes phylum after RH [26]. Previous two large studies conducted in Sweden and China also found that the representation of butyrate-producing bacteria (Roseburia and Faecalibacterium prausnitzii) was lower in type II diabetic patients [27,28]. Gut microbiota transplantations from lean donors to recipients with metabolic syndrome have shown to increase Roseburia and butyrate levels together with improved insulin sensitivity, thus suggesting the importance of butyrate-producing bacteria for blood glucose regulation in humans [29].

There were several limitations in our study. First, the underlying disease, CRC which led to partial colectomy in our study was reported to be associated with metabolic syndrome [30]. However, subjects with diabetes mellitus before colectomy were excluded during enrollment in this study. Furthermore, there were no significant differences in the serum fasting glucose and occurrence of metabolic syndrome between the LAR group and control group. Second, increasing evidence suggests that the gut microbiota composition is altered in patients with CRC [31]. In our cross-sectional study, we lacked the data in baseline gut microbiota status and metabolic profile before surgery to compare with those of post surgery, we cannot clearly distinguish the changes of gut microbiota and metabolic profiles which are caused by partial colectomy or caused by the early alterations of the microbial composition before surgery. Third, the function of the identified bacteria is almost unknown in our study and we did not have metatranscriptomic data, metabolomics, and SCFA measurements. Fourth, we acknowledge that an important drawback of our study is the relatively small number of patients. Fifth, our study analyzed the gut microbiota in subjects who did not consume precisely controlled diets. However, it is widely accepted that diet has an important influence on the composition of human gut microbiota [32]. Furthermore, it is obvious that CRC and metabolic diseases including type II diabetes are intimately linked to diet [33,34].

## Conclusions

Curative partial colectomy with RH for CRC was associated with long term metabolic changes with higher serum fasting glucose and higher occurrence of metabolic syndrome. In parallel with metabolic change, patients with RH showed dysbiosis with a tendency to decreased richness and a significant decrease in the diversity of gut microbiota.

## Supporting information

S1 Table Evolution of library size after sequencing. Number of reads are presented after different filtering steps in the RH, LAR, and control groups.
(XLS)

S2 Table Bacterial community diversity indices. Microbial diversity and richness of gut microbiota (Chao richness estimator, Fisher's alpha diversity index and the Shannon diversity index (SI)) in the RH, LAR, and control groups.
(XLS)
